# Supplementary figures and images for: Mesenchymal Stem Cell Exosomes Enhance Posterolateral Spinal Fusion in a Rat Model
Source: Cells. 2024 Apr 29;13(9):761. doi: 10.3390/cells13090761 (PMC11083285; doi:10.3390/cells13090761)

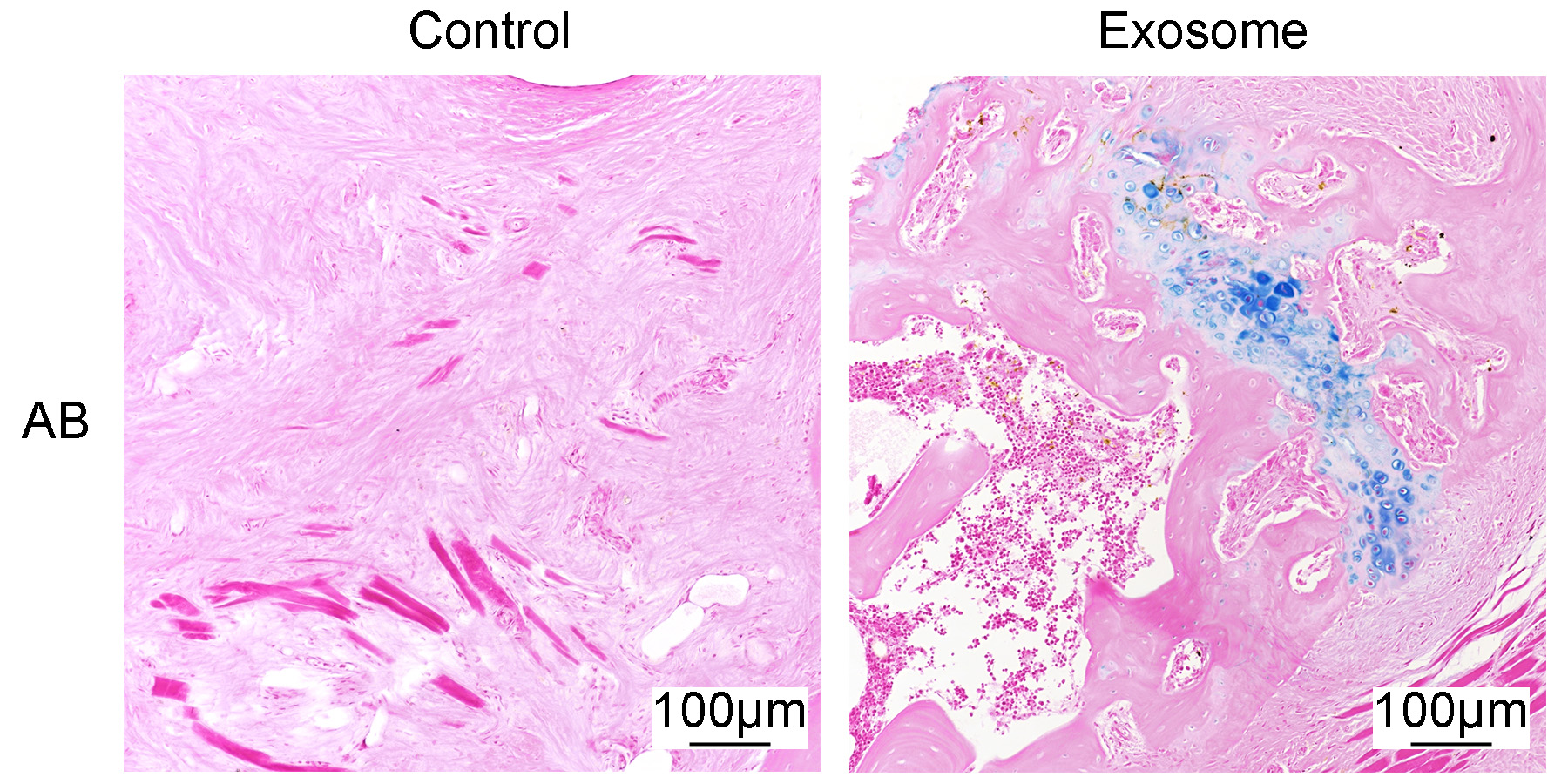

Supplement: Supplementary file 1 [file cells-13-00761-s001.zip › Supplementary files/Figure S3.jpg]

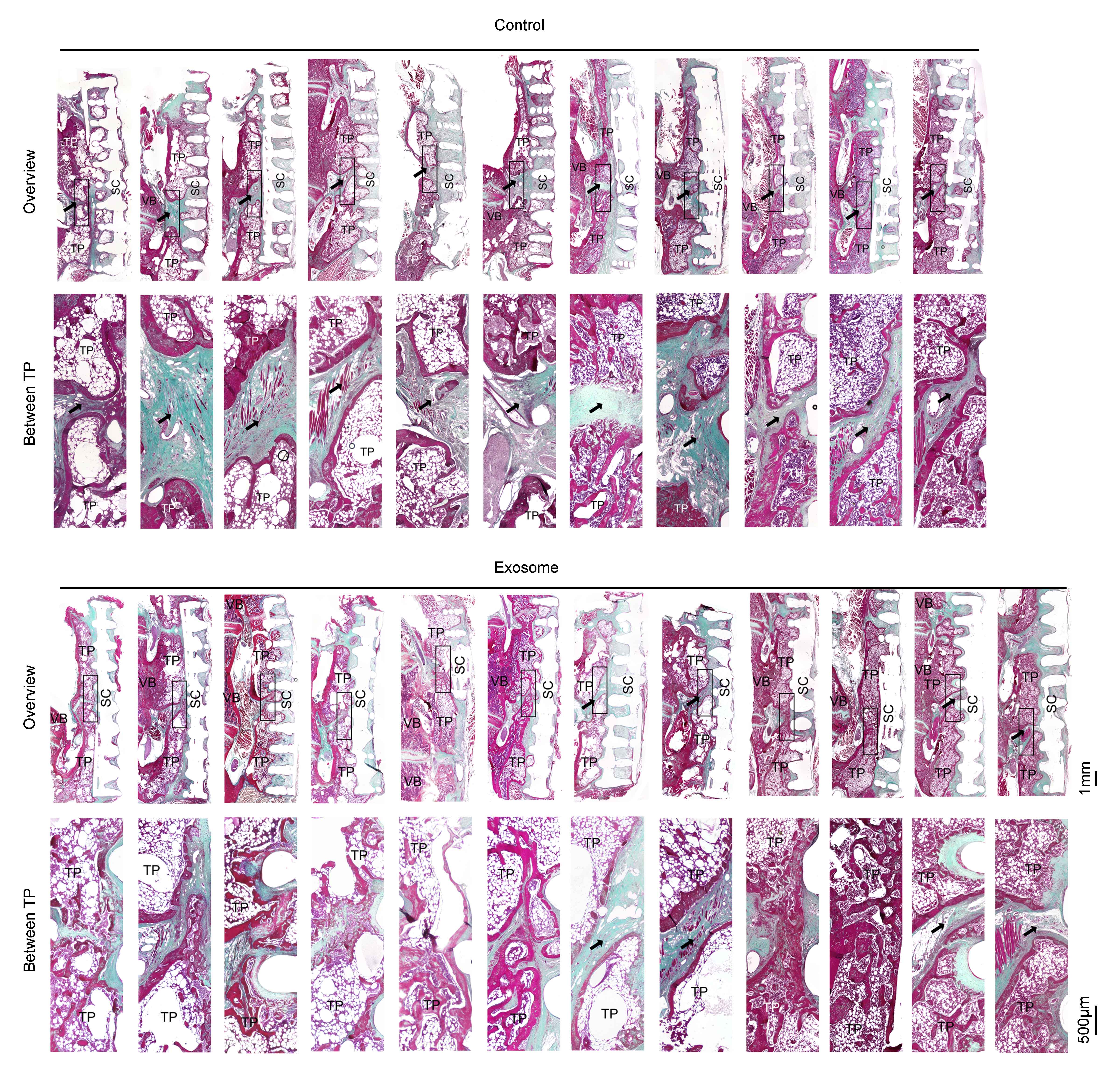

Supplement: Supplementary file 1 [file cells-13-00761-s001.zip › Supplementary files/Figure S2.jpg]

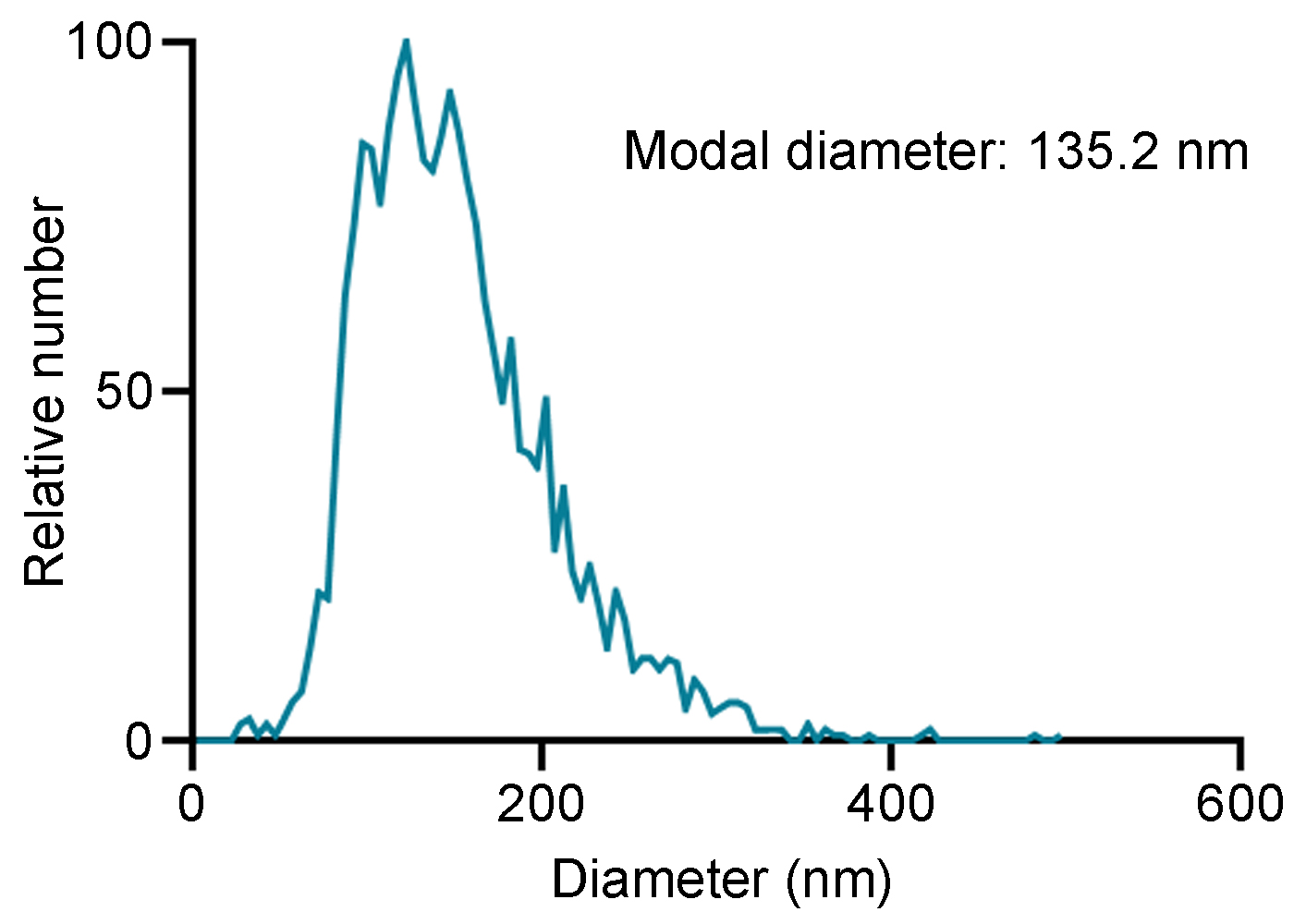

Supplement: Supplementary file 1 [file cells-13-00761-s001.zip › Supplementary files/Figure S1.jpg]
